# A Circadian Clock in Antarctic Krill: An Endogenous Timing System Governs Metabolic Output Rhythms in the Euphausid Species *Euphausia superba*

**DOI:** 10.1371/journal.pone.0026090

**Published:** 2011-10-07

**Authors:** Mathias Teschke, Sabrina Wendt, So Kawaguchi, Achim Kramer, Bettina Meyer

**Affiliations:** 1 Laboratory of Chronobiology, Charité Universitätsmedizin Berlin, Berlin, Germany; 2 Department of Environment and Heritage, Australian Antarctic Division, Kingston, Australia; 3 Antarctic Climate and Ecosystems Co-operative Research Centre, Hobart, Australia; 4 Scientific Division Polar Biological Oceanography, Alfred Wegener Institute for Polar and Marine Research, Bremerhaven, Germany; Yale School of Medicine, United States of America

## Abstract

Antarctic krill, *Euphausia superba*, shapes the structure of the Southern Ocean ecosystem. Its central position in the food web, the ongoing environmental changes due to climatic warming, and increasing commercial interest on this species emphasize the urgency of understanding the adaptability of krill to its environment. Krill has evolved rhythmic physiological and behavioral functions which are synchronized with the daily and seasonal cycles of the complex Southern Ocean ecosystem. The mechanisms, however, leading to these rhythms are essentially unknown. Here, we show that krill possesses an endogenous circadian clock that governs metabolic and physiological output rhythms. We found that expression of the canonical clock gene *cry*2 was highly rhythmic both in a light-dark cycle and in constant darkness. We detected a remarkable short circadian period, which we interpret as a special feature of the krill's circadian clock that helps to entrain the circadian system to the extreme range of photoperiods krill is exposed to throughout the year. Furthermore, we found that important key metabolic enzymes of krill showed bimodal circadian oscillations (∼9–12 h period) in transcript abundance and enzymatic activity. Oxygen consumption of krill showed ∼9–12 h oscillations that correlated with the temporal activity profile of key enzymes of aerobic energy metabolism. Our results demonstrate the first report of an endogenous circadian timing system in Antarctic krill and its likely link to metabolic key processes. Krill's circadian clock may not only be critical for synchronization to the solar day but also for the control of seasonal events. This study provides a powerful basis for the investigation into the mechanisms of temporal synchronization in this marine key species and will also lead to the first comprehensive analyses of the circadian clock of a polar marine organism through the entire photoperiodic cycle.

## Introduction

Antarctic krill (*Euphausia superba* Dana), a small shrimp-like crustacean species, shapes the structure of the Southern Ocean ecosystem due to its central position as direct link between primary producers and apex predators. A decline in winter sea ice duration caused by the climatic warming resulted in a long-term decline in krill biomass in the Scotia Sea sector of the Southern Ocean [1]. An increasing krill fishery might in addition impact the krill stocks in this region [2]. Krill's central position in the food web, the ongoing environmental changes in its habitat, and increasing commercial interest on this species emphasize the urgency of understanding the adaptability of krill to its environment. Krill has evolved rhythmic physiological and behavioral functions which are synchronized with the cyclic changes of the Southern Ocean ecosystem. These functions occur over a daily cycle, such as diel vertical migration (DVM), which is believed to allow krill to maximise food intake in the upper water column during the night and minimise predator risk by migrating in the deep during the day [3]. In addition, seasonal cycles of metabolic regulation [4] and maturity [5] in krill are synchronized with the extreme seasonal cycles in environmental factors such as day length, sea ice extent and food availability. The mechanisms leading to these rhythms are essentially unknown. They are, however, crucial to predict the response of krill to the ongoing environmental changes and, due to its central position, to predict alterations in biodiversity composition and productivity in the Southern Ocean ecosystem. Although it has been speculated that the synchronization between krill and its environment depend upon an internal timing system [3], [6], due to difficulties in maintaining krill in experimental conditions for longer periods the presence of such a system in krill has not yet been demonstrated.

In general, most organisms entrain to the day-night cycles, caused by the earth's rotational movements, by developing an endogenous timing system – a circadian clock (“*circa dies*”: approximately a day) - that allows synchronization of metabolism, physiology and behaviour with the environment and that also may modulate photoperiodic responses. Circadian clocks that have been previously identified are conceptually based upon a similar molecular makeup: transcriptional/translational feedback loops involving rhythmic clock gene expression generate ∼24-hour rhythms on a molecular level [7] that characteristically persist in the absence of external time cues. A molecular framework describing the circadian clock can be exemplified for the fruit fly *Drosophila melanogaster* [8]. At the core of the clock there are key negative components, such as *period* (*per*) and *timeless* (*tim*) and key positive components, such as *clock* (*clk*) and *cycle* (*cyc*). Entrainment of the *Drosophila* clock is mediated through the blue light photoreceptor Cryptochrome (CRY) by promoting the light-dependent degradation of TIM [9]. Therefore, CRY can adapt the transitional oscillations of PER and TIM interactions to changes in light-dark phases. Beside the *cryptochrome* gene that encodes for the light-sensitive photoreceptor in *Drosophila* (insect *cry*1), a second *cry* gene (insect *cry*2) have been identified in some other insect species that encodes for a light-insensitive protein more similar in sequence to mammalian CRYs that act as transcriptional repressors [10].

Crustaceans are a species-rich and highly diverse group of organisms and data on circadian rhythmicity have been reported in some species. However, knowledge about crustacean clock genes is scarce with respect to distribution, oscillatory activity, and chronobiological function [11] and information about the functioning of circadian clocks in polar marine crustaceans is missing.

Here, we performed long-term laboratory experiments with live krill at a specialized experimental aquarium with different simulated light conditions to investigate whether krill possesses a circadian clock and whether such a clock controls key physiological processes of krill. To this end, we investigated temporal mRNA expression levels of the canonical clock gene *cry*2 [6] in individual krill, that were maintained both under a light-dark cycle and constant darkness. In addition, we tested whether gene expression of metabolic key enzymes in these krill show daily or circadian oscillations, and to what extent transcriptional oscillations of these enzymes also persist at the protein-activity level. Finally, we determined oscillatory rhythms at the organismic physiological level in krill by measuring temporal profiles of oxygen consumption. This study presents the first report of a circadian timing mechanism in Antarctic krill and its link to metabolic key processes. Thereby, it provides new insights into the mechanisms of temporal synchronisation of this key species to its challenging environment. This may open a door for further studies dealing with the role of the krill's circadian clock in seasonal time-keeping and in the response of krill to its changing polar environment.

## Results and Discussion

Evidence for the existence of clock genes in crustaceans, their oscillatory rhythms and implications in circadian functions is still patchy. For some time only products of *clock*-, *period*-, and *cryptochrome*-genes have been identified in some freshwater decapod species [12], [13], [14]. This stands in contrast to a wide range of circadian rhythms that are well documented for processes such development, locomotor activity, sensory, and various physiological parameters in many crustacean species [11]. Due to the inaccessibility of high latitudes and the difficulties in maintaining polar marine species in experimental conditions for longer periods little is known for Antarctic krill. Recently, for the first time a mammalian-like *cry* gene that clusters with the insect Cry2 family was identified in krill [6]. The authors observed daily changes in krill *cry*2 mRNA throughout a 24 h cycle and proposed an endogenous circadian time-keeping system in krill that was also previously assumed in a laboratory study of circadian behavioral patterns of krill [3].

To test for the existence of a krill circadian clock, we measured transcript levels of *cry*2 using quantitative PCR on krill samples collected during two independent time course experiments in 2008 and 2010. We used whole heads of krill for our analyses which we expected to include putative oscillator regions such as the retina, the eyestalk system and different neuronal brain structures. In insects, the circadian pacemakers are located in the brain, associated with the visual system and similar molecular elements of the circadian clock system can be expected between crustaceans and insects because of the close phylogenetic relationships, which were recently revealed by several phylogenetic studies [15].

**Figure 1:**
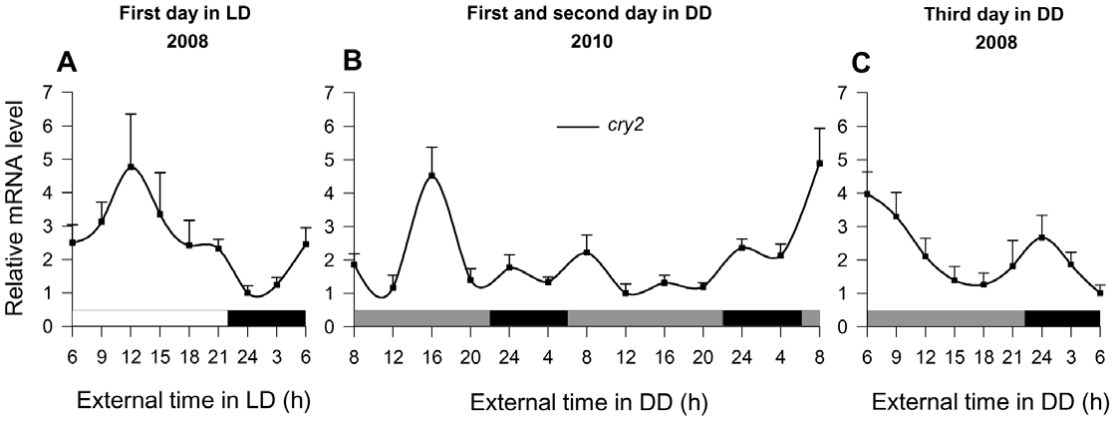
Oscillatory rhythms of *cry*2 in krill. Transcript levels of *cry*2 in krill heads were measured by quantitative PCR from two independent time course experiments, in 2008: Full 24 hour cycle under (**A**) light-dark (LD) conditions and (**C**) at the third consecutive day in constant darkness (DD), and 2010: Full 48 hour cycle at the first and second consecutive day in DD (**B**). Significant oscillations were observed in LD conditions (24 h period with p<.01, CircWave see material and Methods) and at the third day in DD (∼18 h period with p<0.05). Fisher's LSD post-hoc analysis of *cry*2 oscillatory rhythms indicate the following significant (p<0.05) differences among time points: *cry*2 expression under LD conditions at 9:00, 12:00, and 15:00 h was significantly elevated compared to expression at 6:00, 18:00, 21:00, 24:00, and 3:00 h, and expression at 24:00 and 3:00 h was significantly reduced compared to expression at 6:00, 9:00, 12:00, 15:00, 18:00, and 21:00 h; *cry*2 expression during the first day in DD at 16:00 h was significantly elevated compared to expression at 8:00, 12:00, 20:00, 24:00, and 4:00 h, and expression at 8:00 h (second time point) was significantly elevated compared to expression at 12:00, 20:00, 24:00, and 4:00 h; *cry*2 expression during the second day in DD at 8:00, 24:00 and 4:00 h was significantly elevated compared to expression at 12:00, 16:00, and 20:00 h, and expression at 8:00 h (second time point) was significantly elevated compared to expression at 8:00 (first time point), 12:00, 16:00, 20:00, 24:00 and 4:00 h; *cry*2 expression during the third day in DD at 6:00 (first time point), 9:00, and 24:00 h was significantly elevated compared to expression at 12:00, 15:00, 18:00, 21:00, 3:00, and 6:00 h (second time point). White and black bars refer to light and dark periods. Grey bars refer to the previous light periods. Shown are means ± SEM (n 3–7).

On the basis of a first set of laboratory experiments with live krill in 2008 we found that expression of *cry*2 was highly rhythmic both in a light-dark (LD) cycle and in constant darkness (DD; Figure 1A and C). *Cry*2 transcripts showed significant daily oscillations (24 h with p<0.01) with a peak-to-trough ratio of ∼5. The expression was significantly upregulated around the middle of the light phase with a peak around 12:00 h (p<0.05; Figure 1A) and lowest amounts around two hours after lights-off (24:00 h). Our observation of daily *cr*y2 oscillation corresponds to that reported for krill caught from the wild [6] where *cry*2 expression levels in krill were found to be highest throughout the day. Under constant conditions, we found that even on the third day after the light regime was changed from the LD cycle to DD *cry*2 showed a significant circadian oscillation (p<0.05; peak-to-trough ratio of ∼4). *Cry*2 expression was significantly upregulated around 6:00 h at the beginning of the subjective day and around 24:00 h early in the subjective night (p<0.05; Figure 1C). We found a minimum of *cry*2 expression around 15:00–18:00 h late in the subjective day and again around 6:00 h at the end of the subjective night.

Overall, also considering the inhomogeneity of our krill samples (both in genotype and developmental stage), this strongly suggests that krill possesses an endogenous circadian clock. To our knowledge this is the first evidence for circadian rhythmicity of a mammalian type *cry* gene in any crustacean species. The only previous evidence for rhythmicity of a *cry* gene in crustaceans was obtained from western blot analyses of a *Drosophila* like Cry protein in the brain of the crayfish *Procambarus clarkii* [13]. Likewise to our findings of *cry*2 mRNA levels, a daily rhythmic pattern of Cry abundance was observed under a 12:12 LD cycle with highest abundance throughout the day and a minimum in the middle of the night. However, the temporal expression of *cry*2 has been previously investigated in two insect species, the honeybee *Apis mellifera* [16] and the monarch butterfly *Danaus plexippus* [17]. Both species showed marked daily and circadian rhythms in *cry*2 mRNA levels, but with elevated expression during the dark (or subjective dark) phase and reduced expression during the light (or subjective light) phase.

Surprisingly, the endogenous period in DD seems to be much shorter than 24 hours (∼18 h with p<0.05; Fig. 1C) which could account for the differences in the relative phases of *cry*2 mRNA oscillations comparing LD and DD conditions. To get a better picture of the endogenous period under DD conditions we analyzed a second independent time course that was conducted in 2010 and covers the first and second consecutive days in DD (Fig. 1B). Although we found no significant periodicity (18–24 h with p  =  0.1) within the first two days in DD, *cry*2 expression was significantly upregulated during the middle of the first subjective day, but with expression peaking 4 hours later at 16:00 h (p<0.05; peak-to-trough ratio of ∼4.5). *Cry*2 mRNA level at 12:00 h, previously showing the highest value under LD conditions, now showed significantly the lowest value (p<0.05). Consistent with the much shorter circadian period than 24 hours, a different oscillatory pattern was observed during the second day in DD. *Cry*2 expression level at 8:00 h, although only representing a 2 fold increase, was significantly elevated from 4:00 h and 12:00 h (p<0.05). After reaching the significantly lowest value at 12:00 h (p<0.05), no clear peak could be detected until the end of the second subjective day. After that, expression level started to increase until the end of the second subjective night and a maximum was reached at the beginning of the third subjective day (8:00 h with p<0.05), when *cry*2 expression was significantly upregulated. Interestingly, this peak is consistent with the *cry*2 expression level at 6:00 h that what was previously found to be significantly upregulated during the independent time course experiment in 2008. When inspecting the two independent time courses in DD together (keeping the limitations in mind combining independent experiments), we then found a significant circadian profile with a period of ∼18 hours (p<0.05, see Fig. S1). However, to get a better estimation of the circadian period of clock gene expression in krill, a longer, continuous time series with a higher sampling rate is required.

Which adaptive value might a short circadian period have in the evolution of krill? Since not the free-running period (i.e. the genotype) but the phase of entrainment (i.e. the phenotype) is under evolutionary selection pressure [18], we speculate that the circadian system of krill has to delay every day for several hours to keep a stable phase of entrainment in the light-dark environment. This would imply that the circadian system of krill probably has a high-amplitude phase-response curve for light [19] and, consequently, a wide range of entrainment [20]. The biological meaning of our speculation of such a putative wide range of entrainment might lay in the fact that Antarctic krill probably needs to be able to entrain to much more extreme photoperiods (that in the area of main distribution of krill range from ∼21 hours of light in summer to ∼3 hours of light in winter) than animals living in less extreme latitudes. For example, many rodents cannot stably entrain to photoperiods that are below 6 hours of light or are beyond 18 hours of light [19], which we believe is correlated with their relatively narrow range of entrainment. The circadian system of Antarctic krill, however, evolved in more extreme photoperiods and might have found a means to stably entrain to these photoperiods by developing a circadian system with a wide entrainment range. Indeed, Aschoff and Pohl described already in 1978 [21] that, due to a weakening of *Zeitgeber* strength, in extreme photoperiods entrainment is more difficult to achieve than in symmetrical light-dark cycles. This, in turn, implies that the entrainment region needs to be wider under extreme photoperiods. Moreover, Pittendrigh and Daan found that the very *circadian* nature of the free-running period (i.e. its deviation from 24 hours) is a feature of circadian clocks that helps to stably entrain the circadian system throughout the year when photoperiod changes [22] – a property which might have evolved in krill in an extreme form.

Future experiments and analyses are necessary to get a better picture of the circadian oscillator in krill. In order to better understand the functioning of the circadian clock in krill it will be necessary to identify other canonical clock genes such as *period*, *timeless*, *clock and cycle*. The cloning of the full-length sequences of some of these genes is currently in progress and, together with accompanying analyses, is going to be published in the near future.

**Figure 2:**
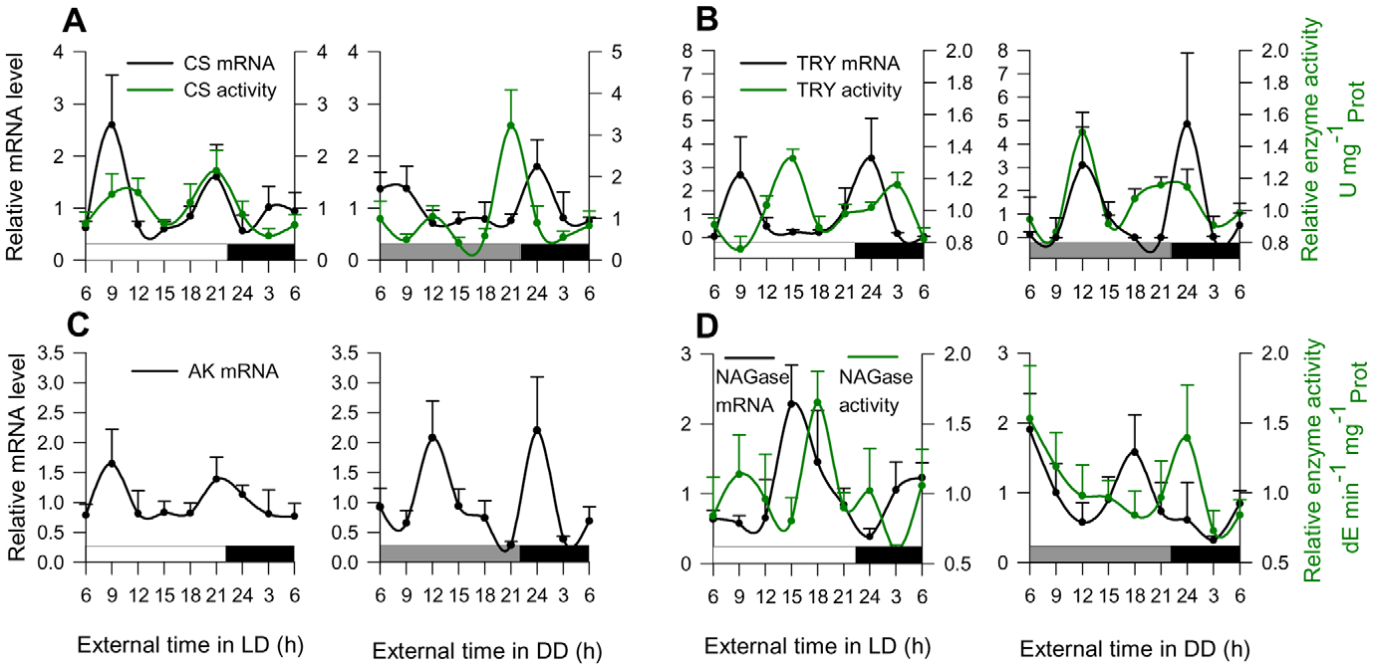
Oscillatory rhythms of metabolic key enzymes in krill. Transcript levels and enzyme activity of metabolic key enzymes were measured under light-dark (LD) conditions and at the third consecutive day in constant darkness (DD; 24 hour time course experiment in 2008, see Material and Methods). (**A**) Citrate synthase (CS), (**B**) trypsin (TRY), and (**C**) aldo-keto reductase (AK) show bimodal circadian oscillations (∼9–12 h period) in transcript abundance (LD; p<.05 for *cs*, p<0.01 for *try*, p<0.05 for *ak*, DD; p<.01 for *try*, p<0.01 for *ak*, CircWave see Material and Methods) and enzymatic activity (LD; p<0.01 for CS, p<0.001 for TRY, DD; p<0.05 for CS, p = 0.05 for TRY). No enzyme activity assay was available for AK. Note that CS, TRY and AK oscillations show a similar phase relationship which is in sharp contrast to (**D**) β-N-acetylglucosaminidase (NAGase). White and black bars refer to light and dark periods. Grey bars refer to the previous light periods. Shown are means ± SEM (n = 3–7).

To test whether metabolic functions in krill are under circadian regulation, we measured the expression and the activity of key metabolic enzymes. Citrate synthase (CS), trypsin (TRY), aldo-keto reductase (AK) and β-N-acetylglucosaminidase (NAGase) all showed circadian oscillations in transcript abundance and enzyme activity (Figure 2). Interestingly, CS, TRY, and AK showed oscillatory patterns with roughly 9–12 h period under both lighting conditions (Figure 2A, B and C). About 12 h rhythms of behaviour and physiology in crustaceans have been previously described. The Norway lobster *Nephrops norvegicus* for instance shows both 24 h and 12 h rhythms in locomotor activity that persists in constant darkness [23]. Likewise, the crayfish *P. clarkii* shows a 12 h rhythm of spontaneous locomotion that also persists in the free run [24]. Gaten et al. [3] identified a 12 h rhythmic component in the DVM of Antarctic krill during laboratory experiments which correlates with findings from the field [25], where a 12 h component in DVK of krill becomes predominant at certain times during the year when food is scarce. Often, these rhythms have been interpreted as bimodal patterns of one circadian-behavioral output.

Considering the functional relevance of the bimodal rhythms in this study, it is notable that CS, TRY and AK oscillations showed a similar phase relationship which was in sharp contrast to NAGase (Figure 2D). This suggests a common functional trait of the former three enzymes and may reflect the fact that CS, TRY and AK are directly or indirectly related to carbohydrate metabolism. AK is involved in glycolysis, the initial process of carbohydrate metabolism. CS catalyzes reactions in the citric acid cycle, a metabolic pathway also involved in the chemical conversion of carbohydrates downstream the glycolysis. TRY is the dominant proteinase in Antarctic krill playing a major role in the digestive system of this species, and a strong positive correlation between digestive proteases and carbohydrases was already found [26]. In some crustaceans carbohydrate metabolism is known to be under circadian control [27], [28] and often shows bimodal circadian rhythms. In the crayfish *P. clarkii* for instance, eyestalk contents of the Crustacean Hyperglycaemic Hormone (CHH), which plays a role in the regulation of carbohydrate substrates, show a bimodal circadian rhythm that coincides with bimodally increasing haemolymph glucose levels [29]. There is also evidence from laboratory experiments that Antarctic krill shows bimodal circadian rhythms in the level of glucose in its haemolymph and hepatopancreas [27]. These observations of a bimodal pattern of carbohydrate metabolism in krill almost coincide with the present bimodal oscillatory patterns in transcript abundance and enzyme activity of metabolic key enzymes that are associated with this functional trait.

**Figure 3:**
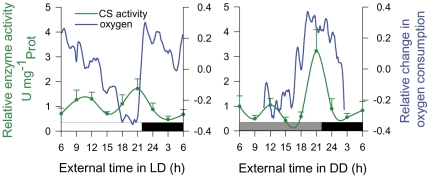
Oscillatory rhythms of oxygen consumption in krill. Temporal profiles of oxygen consumption of individual krill were determined under light-dark (LD) conditions and at the third consecutive day in constant darkness (DD; 24 hour time course experiment in 2008, see Material and Methods). Oxygen consumption shows ∼9–12 h oscillations that correlate with the temporal activity profile of citrate synthase (CS). White and black bars refer to light and dark periods. Grey bars refer to the previous light periods. Shown are means ± SEM (n = 3–7).

Since CS is a key enzyme of aerobic energy metabolism, rhythms of CS activity should lead to corresponding temporal changes in oxygen consumption. This is indeed what we observed (Figure 3), even though, ∼12 h bimodal behavioral rhythms (e.g. locomotor activity) of krill, previously observed in the field and in the laboratory [3], may also have contributed to these oscillations. A link between carbohydrate metabolism and locomotor activity was previously shown in some crustacean species, when high levels of glucose in the haemolymph correlated with phases of high locomotor activity [28].

In contrast, the key enzyme NAGase is not associated with carbohydrate metabolism. NAGase is a key enzyme in the moulting process of krill and other invertebrates and is needed to degrade the chitin microfibers of the cuticle. This could account for the different oscillatory pattern of NAGase compared to the three other enzymes which were associated with carbohydrate metabolism, indicating a different mode of circadian regulation. Indeed, the oscillatory rhythms in transcript abundance and enzyme activity that were observed for NAGase showed a pattern more similar to what was found for the oscillatory rhythms of clock gene expression rather than a pronounced 12 h rhythm (see Figure 1 A,C and Figure 2D).

Overall, these results suggest that the krill endogenous circadian clock governs metabolic and physiological output rhythms which, in the case of CS, TRY, and AK, we interpret as evidence of a circadian bimodal output. Different oscillatory patterns among enzymes may reflect independent functional traits. Since we cannot formally exclude the possibility that these bimodal circadian rhythms are regulated by mechanisms other than or in addition to the circadian clock, further studies will be necessary to investigate the contribution of the circadian clock to rhythmic variations in expression and activity of metabolic enzymes in krill and thereby will give a better understanding of how rhythmic physiology and behavior in krill will be regulated.

In conclusion, we present the first report of an endogenous circadian timing system in Antarctic krill and its link to metabolic key processes. We suggest that this system is of essential importance for krill as it facilitates synchronization of its physiology and behaviour to daily environmental cycles. However, krill's circadian clock may not only be critical for synchronization to the solar day but also for the control of seasonal events, such as reproduction and metabolic depression. In general, our understanding of how circadian clocks of high latitude organisms such as krill might have adapted to the strong variability in annual day length that at the extreme may ranges from constant darkness in winter to constant light in summer is small. For the Arctic reindeer *Rangifer tarandus* overt circadian rhythmicity of locomotor activity was only observed under pronounced LD cycles as present during the Arctic autumn and spring, indicating a damped circadian clock during periods of continuous light in summer or continuous darkness in winter [30] as also confirmed by other experimental observations [31]. Conversely, some other Arctic species such birds [32], squirrels and porcupines [33] suggesting an intact circadian clock during the Antarctic summer by maintaining circadian activity cycles. These results may indicate entrainment of the circadian clock by other environmental rhythmic cues than changes in day length. Indeed, Pohl [34] could show that regular daily changes in spectral composition of sunlight can act as *Zeitgebers* for the synchronisation of living rhythms for high latitude organisms living in continuous daylight. These results suggest that polar organisms might show a wide plasticity of the circadian clock machinery that enable them to cope with prolonged periods without the presence of a strong *Zeitgeber* either by adopting arrhythmic behaviour or by switching to alternative environmental cues . This study provides the basis for investigating the mechanisms of temporal synchronization in krill and will also lead to the first comprehensive analyses of the circadian clock of a polar marine organism through the entire photoperiodic cycle. Understanding the mechanisms of temporal synchronization of krill to its environment will be of essential importance for predicting the response of this key species to its changing environment.

## Materials and Methods

### Ethics Statement

All animal work has been conducted according to relevant national and international guidelines. Krill catches, welfare and experimentation were based on permission of the Department of Environment and Heritage (DEH) of the Australian Government and were conducted in accordance with the Antarctic Marine Living Resources Conservation Act 1981 (AMLR, permit number: 06_09_2220) and the Environment Protection And Biodiversity Conservation Act 1999 (EPB, permit number: WT2007-1480).

### Animals

*E. superba* were caught by oblique hauls of several Rectangular Midwater Trawls, using a pelagic net (RMT 8), in the upper 200 m of the water column. Catches were made in East Antarctica (between 65° 19′ S, 125° 37′ E, 17 Sep 2007 and 64° 08′ S, 119° 16′ E, 09 Oct 2007) during the voyage V1 07/08 with RSV *Aurora Australis*. Immediately after hauling, krill were transferred as quickly as possible into 200 L tanks located in a temperature constant room at 0°C and dim light. Each day 50% of the water was exchanged with fresh pre-chilled seawater to ensure a continual turnover of food and nutrients. Twice a day, dead animals and moults were removed from the tanks [35]. After arriving in Hobart, Tasmania (17 Oct 2007), krill were delivered directly to the Australian Antarctic Division (AAD) aquarium and kept in a 1670 L holding tank. A detailed description of the holding tank system at the AAD and of krill maintenance in the laboratory is described elsewhere [36], [37].

### Maintaining krill in the laboratory

The present study is based on two experiments which were conducted in 2008 and 2010. The first experiment was started on 07 Feb 2008 by separating 340 krill of mixed sex (mean length ∼38 mm) from the holding tank into two cylindrical 100 L tanks (170 krill each) situated within one 1000 L rectangular container. This system was connected to a 5000 L chilled sea water recirculation system of the aquarium. The water was maintained at 0.5°C. All tanks within the container had separate water in- and outflow. The chilled water was simultaneously pumped into the container and into each experimental tank. All tanks drained back to the container from where the water entered into an array of filtration devices. After filtration the water was pumped back to the container and tanks. The design of the experimental re-circulating facility guaranteed identical water quality and temperature for every experimental stock throughout the study [35].

Each tank was covered with a black lightproof plastic container with a sliding door at the front side to create a separate light compartment. Lighting was provided by fluorescent tubes (Osram L18W/640 Cool White) covered with a filter film around the outside (ARRI, Marine Blue 131). Photoperiod and light intensity were controlled by a PC-controlled timer system. The two experimental tanks were exposed to a light:dark (LD) regime of 16 hours light and 8 hours darkness with lights-on and lights-off at 6:00 h and 22:00 h external time, respectively. Corresponding to 1% light penetration to 30 m depth a maximum of 100 lux light intensity was set at the surface of the tanks during midday. Prior to the collection of samples all specimens in both experimental tanks were exposed for a period of 3–4 weeks to these conditions to provide a successful acclimatisation to the aquarium conditions. Both experimental stocks were fed daily with the same algae used in the holding tank at final densities of 3.8×10^4^ cells mL^−1^ for *Phaeodactylum tricornutum*, 9.2×10^4^ cells mL^−1^ for *Isochrysis sp.* and 6.6×10^4^ cells mL^−1^ for *Pavlova sp*. Animals were fed at random times during the day to avoid a feeding pattern becoming a potential entrainment cue.

The second experiment was started on 15 Feb 2010 by separating 150 krill of mixed sex (mean length ∼38 mm) from the holding tank into one cylindrical 100 L tanks situated within one 1000 L rectangular container. Maintenance of krill and the design of the experimental facility were identical to what was described above. The experimental tank was exposed to a LD regime of 16 hours light and 8 hours darkness with lights-on and lights-off at 6:00 h and 22:00 h external time, respectively.

### Experimental design

For analyzing rhythmic gene expression two 24 hour collection series were conducted in the first experiment. At 05 Mar 2008 a first time series was sampled in experimental tank I under LD 16∶8 conditions. Krill were sampled at regular intervals (every three hours) over a full 24 hour cycle. Animals were taken at 6:00, 9:00, 12:00, 15:00, 18:00, 21:00, 24:00, 3:00 and 6:00 h external time. After the light regime was changed in experimental tank II from the LD cycle to constant darkness (DD) on 10 Mar 2008, a second 24 hour collection series was performed at the third day in DD (12–13 Mar 2008). Krill were sampled at 3 hour intervals at 6:00, 9:00, 12:00, 15:00, 18:00, 21:00, 24:00, 3:00 and 6:00 h external time.

For molecular analyses 10 animals were sampled at each time point, immediately frozen in liquid nitrogen and stored at −80°C. Sampling in darkness was carried out under dim red light. Heads of frozen krill were used for quantitative real-time PCR analyses to investigate the temporal expression patterns of *cry*2 and metabolic key enzymes. Abdominal segments of the identical krill were used to measure corresponding enzyme activities. For determining changes in the overall metabolic activity of krill under the different light regimes temporal profiles of oxygen consumption of individual krill were determined in LD 16:8 (experimental Tank I) and in DD (experimental tank II) for at least 39 hours.

In 2010, after the light regime was changed in the experimental tank from the LD cycle to DD on 17 Mar 2010, a 48 hour collection series was performed at the first and second consecutive day in DD (17–19 Mar 2010). Krill were sampled at 4 hour intervals at 8:00, 12:00, 16:00, 20:00, 24:00, 4:00, and 8:00 h external time. For molecular analyses 10 animals were sampled at each time point, immediately frozen in liquid nitrogen and stored at −80°C. Sampling in darkness was carried out under dim red light. Heads of frozen krill were used for quantitative real-time PCR analyses to investigate the temporal expression patterns of *cry*2.

### Quantitative RT-PCR

#### Primers

Primers for qPCR analysis were designed around sequences of interest using PrimerQuest^SM^ (Integrated DNA Technologies) to generate PCR products of between 100 and 200 bp (Table 1). The primer set for krill *cry*2 was designed around the full length gene sequence as previously published [6], (GenBank: FM 200054.1). Primer sets for metabolic key enzymes *ATP-citrate synthase* (*cs*), *trypsin* (*try*), *aldo-keto reductase* (*ak*), *β-N-acetyglucosaminidase* (*nag*) and a refernce gene *phosphoenol-pyruvatecarboxykinase* (*pep-ck*) were designed against Expressed Sequence Tags (ESTs) that showed significant homology to known genes: for *cs* see (*Euphausia superba* EST database: KRCO1002, http://krill.cribi.unipd.it), for *try*, *ak*, *nag* and *pep-ck* see (dbEST: FL688135-FL689414, http://www.ncbi.nlm.nih.gov/projects/dbEST and [38]).

**Table 1:** Primer sequences used in qPCR.

Gene name	Primer sequence 5′-3′	Product size (bp)
*cryptochrome*2	F: ATGCAGAAGCTCTTGCCAAATGGGR: AGGAAACATGCAACAGCATGACGG	127
*ATP-citrate synthase*	F: CATGTGCAAATGCTGATGCCGAGAR: ACACACCATTGATCACCACTCCCA	114
*trypsin*	F: ATGCCTATGGTGAGGGTGAGR: GGTAGTTGGGTCTGGCACAT	174
*aldo-keto reductase*	F: TTTCAGATTCAACGCAATGTGR: GCTGCACACTCGACCATTAC	147
*β-N-acetylglucosaminidase*	F: AGTGTTCTGCCGATTTTGGTR: TCCTCAACAGACCCACTTCC	169
*phosphoenolpyruvatecarboxykinase*	F: TGTTGAAGGTAGTGGCCAAAR: GAAACACGGTGTCATGGTTG	138

#### qPCR methodology

A single krill head was used for each RNA extraction and 3–7 independent krill of mixed sex and age were prepared for each time point. Frozen krill heads were cut off on a cooling element behind the eyestalks and were immediately transferred to a mortar and preground in liquid nitrogen to a homogenous powder. The powder was then stored in 1 mL TRIzol® reagent (Invitrogen) and total RNA was extracted according to the supplier's instructions. Two µg of total RNA were reversely transcribed to cDNA using RevertAid™ M-MuLV Reverse Transcriptase (Fermentas). The cDNA was diluted 1∶10 and 8 µL were used as template for qPCR. Expression levels were measured with a SYBRGreen fluorescence assay using 10 µL of SYBRGreen PCR Master Mix (Fermentas) , 2 µL of forward and reverse primers (3 µM) and 8 µL cDNA in a total volume of 20 µL and analysed by a ABI PRISM 7000 detection system (Applied Biosystems). The transcript levels of all genes were normalised against the reference gene *pep-ck* and evaluated according to the 2^−ΔΔCt^ quantification method [39]. Note that *pep-ck*, which was previously found to show stable mRNA levels in a comparative gene expression study in krill [38], showed no temporal oscillations in mRNA abundance when measuring gene expression levels in our experimental krill over a full 24 hour cycle in LD and DD. Hereby, we used the same sampling intervals as for the analyses of the temporal expression patterns of clock genes and metabolic key enzymes.

The used primer sets are shown in Table 1. Dissociation curves were performed for each primer set to confirm the specifity of the amplicon and standard curves were conducted to verify the efficiency of the PCR reaction. Expression levels were then calculated relative to the minimal or mean expression level for each gene and represent the mean ± SEM of 3–7 krill heads. The significance of periodicity and period length for all mRNA oscillations was tested for each day with CircWaveBatch v3.3 software as previously described [40]. Differences among time points for *cry*2 expression levels were additionally analysed by ANOVA followed by a Fisher LSD post-hoc test. The significance level for all analyses was set at p≤0.05.

### Measurements of enzyme activity

#### Tissue preparation

The fifth abdominal segment (AS) of frozen animals was dissected carefully for determination of enzyme activities. All dissections took place on a cooling element to avoid thawing. The AS were homogenised in pre-weighed 2 mL tubes containing ceramic beads of 1.4 and 2.8 mm diameter (Precellys®) in ice-cold deionised water at a concentration of 100 mg fresh weight (fw) mL^−1^, which corresponds to a dilution of 1∶10. Homogenization was performed using the Precellys® 24 homogenizer with two agitation intervals of 15 s at 5000 rpm and one pause of 10 s between intervals. A constant temperature of 4°C within the homogenization chamber was maintained using liquid nitrogen and a Precellys® cooling module. The homogenates were centrifuged for 15 min at 14000 rpm (4°C) and the supernatants were then transferred into new reaction tubes and stored at −80°C until analysis.

### Enzyme assays

Before measuring the activity of the metabolic enzymes, the frozen samples were defrosted gently on ice and centrifuged at 5000 rpm and 4°C for 10 min. The supernatants were used to determine the enzyme activity and the corresponding protein content for calculation of specific enzyme activity (per mg protein, mg _Prot_^−1^). The protein content was measured with the BIO-RAD protein assay, based on the method of Bradford [41].

CITRATE SYNTHASE (EC 2.3.3.1): CS activity was determined according to Stitt [42] using certain modifications as described elsewhere [43]. To a semi-microcuvette filled with 780 µL 0.5 M Tris/HCl-buffer (pH 8, supplemented with 0.1 M KCl and 1 mM EDTA), we added 30 µL DTNB (5,5′-dithiobis(2-nitrobenzoic acid)), 6 mM in buffer, Sigma D8130), 30 µl acetyl-CoA (acetyl coenzyme A tri-lithium salt, 6 mM, Roche Diagnostics 101907) and 30 µL sample. After5 min of incubation at 30°C, the reaction was initiated by adding 30 µL oxaloacetic acid (12 mM, Sigma O4126) and monitored continuously at 412 nm for 3 min. Samples were assayed in triplicate. The activity was calculated as U x mg _Prot_^−1^ ( =  µmol min^−1^ mg _Prot_^−1^) using the extinction coefficient ε_412_  =  13.6 L mmol^−1^ cm^−1^.

TRYPSIN (EC 3.4.21.4): TRY activity was assayed with Nα-benzoyl-DL-arginine-*p*-nitroanilide (l-BAPNA, Merck 1.10754) as substrate [44]. The samples (50 µL) were mixed thoroughly in a cuvette with 930 µL of 0.1 M Tris/HCl buffer, pH 8.0, containing 0.01 M CaCl_2_ and incubated for 5 min at 30°C. The reaction was started by the addition of 20 µL of substrate solution (BAPNA, 0.05 M in DMSO). The substrate concentration in the cuvette was 1 mM. The change of absorbance at 405 nm was recorded for another 3 min at constantly 30°C. Samples were assayed in triplicate. The activity was calculated as U x mg _Prot_^−1^ ( =  µmol min^−1^ mg _Prot_^−1^) using the extinction coefficient ε_405_  =  10.2 L mmol^−1^ cm^−1^.

β-N-ACETYLGLUCOSAMINIDASE (EC 3.2.1.52): NAGase activity was determined by the hydrolysis of 4-nitrophenyl-N-acetyl-β-D-glucosaminide (NP-GlcNAc) as previously described [45], [46]. The reaction mixture contained 200 µL sodium-phosphate buffer (0.1 M, pH 7.5) and 20 µL of the sample. After preincubation for 5 min at 30°C in a thermomixer (Eppendorf, 5437), the reaction was initiated by the addition of 50 µL of NP-GlcNAc solution (Sigma N9376, 5 mM in water). After 45 min of incubation, the reaction was terminated by the addition of 500µL of 1 M Na_2_CO_3_ solution and subsequent cooling on ice. Liberated p-nitrophenol was measured spectrophotometrically at 405 nm. Samples were assayed in triplicate with two blanks for which Na_2_CO_3_ was added prior to the enzyme extract. NAG activity was calculated as the change in absorbance per time and mg _Prot_^−1^ (ΔE_405_ x min^−1^ x mg _Prot_^−1^).

The values of all enzymes were expressed relative to the mean level for each enzyme and are the mean ± SEM of 3 abdominal segments. The significance of periodicity and period length for all enzymatic oscillations was tested with CircWaveBatch v3.3 software as previously described [40].

### Oxygen consumption measurements

Temporal profiles of oxygen consumption of individual krill were determined by measuring the decrease of oxygen saturation in respiration chambers using oxygen microoptodes (PreSens, Neuburg, Germany). We used a microoptode array for parallel operation of five optodes (sensors) to measure oxygen saturation in the water. Each sensor consists of a fiber optic cable connected with a standard glass fiber plug to connect it to a multi-channel optode array. For better protection of the sensor tip, the sensor end is mountained in an air and water tight syringe from where the tip can be inserted into the respiration chamber. All data are transmitted directly to a computer for continuous registration. For a detailed description of the array and technical specifications of microoptodes as provided by the manufacturer see [47] or (www.presens.de).

Oxygen saturation measurements were performed in filtered seawater (0.1 µm pore size). From each experimental tank (LD 16∶8 and DD), four krill were used and incubated individually in 1.5 L respiration chambers (laboratory glass bottles, Schott DURAN®, Germany). One chamber of the same volume without krill was used as control for each tank. The krill were rinsed, added to the incubation bottles, which were then closed with an air and water tight lid, and incubated in the experimental tanks. Each bottle-lid was connected with a syringe, through which the sensor end was inserted into the respiration chamber (see above). Measurements of oxygen saturation started immediately after closing of the respiration chambers and inserting the sensors. Data were recorded every 20 s for at least 39 h. Before starting measurements for each sensor, two-point calibrations were performed with all sensors connected to the same water reservoir. Nitrogen bubbling and air bubbling were used to calibrate the 0% and 100% air saturation points, respectively.

For calculations of temporal changes in oxygen consumption in a respiration chamber, first, linear regressions of oxygen saturation versus time were calculated. Subsequently, a moving average (24 h) was used to smooth out the long-term trend of decreasing oxygen saturation and to demask rhythmic cycles in the oxygen saturation data for each individual krill. Temporal regressions of four krill were then pooled and corrected for oxygen saturation changes inside control chambers then representing mean temporal changes in oxygen saturation relative to the mean level of a 24 h period. Exponential smoothing was applied to the resulting data to produce the plot for presentation. We assumed a simple inverse correlation between oxygen saturation and oxygen consumption and therefore the data were finally expressed as relative changes in oxygen consumption with positive values indicating an increase and negative values indicate a decrease in oxygen consumption.

## Supporting Information

Figure S1 **Antarctic krill exhibit a surprisingly short circadian period.** *Cry*2 expression levels, monitored over 78 hours (combined data from two independent time courses in 2008 and 2010), reveal a significant circadian periodicity with a period of ∼18 hours (p<0.05, CircWave see Material and Methods). Predicted peaks from a fitted 18 h sinusoid (at circadian time (CT) 17, CT 35, CT 53, and CT 71) are correlating well with peak expression of *cry*2 mRNA level in vivo (at CT 16, CT 32, CT 56, and CT 72).
(TIF)
