# Supplementary figures and images for: A Circadian Clock in Antarctic Krill: An Endogenous Timing System Governs Metabolic Output Rhythms in the Euphausid Species Euphausia superba
Source: PLoS One. 2011 Oct 7;6(10):e26090. doi: 10.1371/journal.pone.0026090 (PMC3189233; doi:10.1371/journal.pone.0026090)

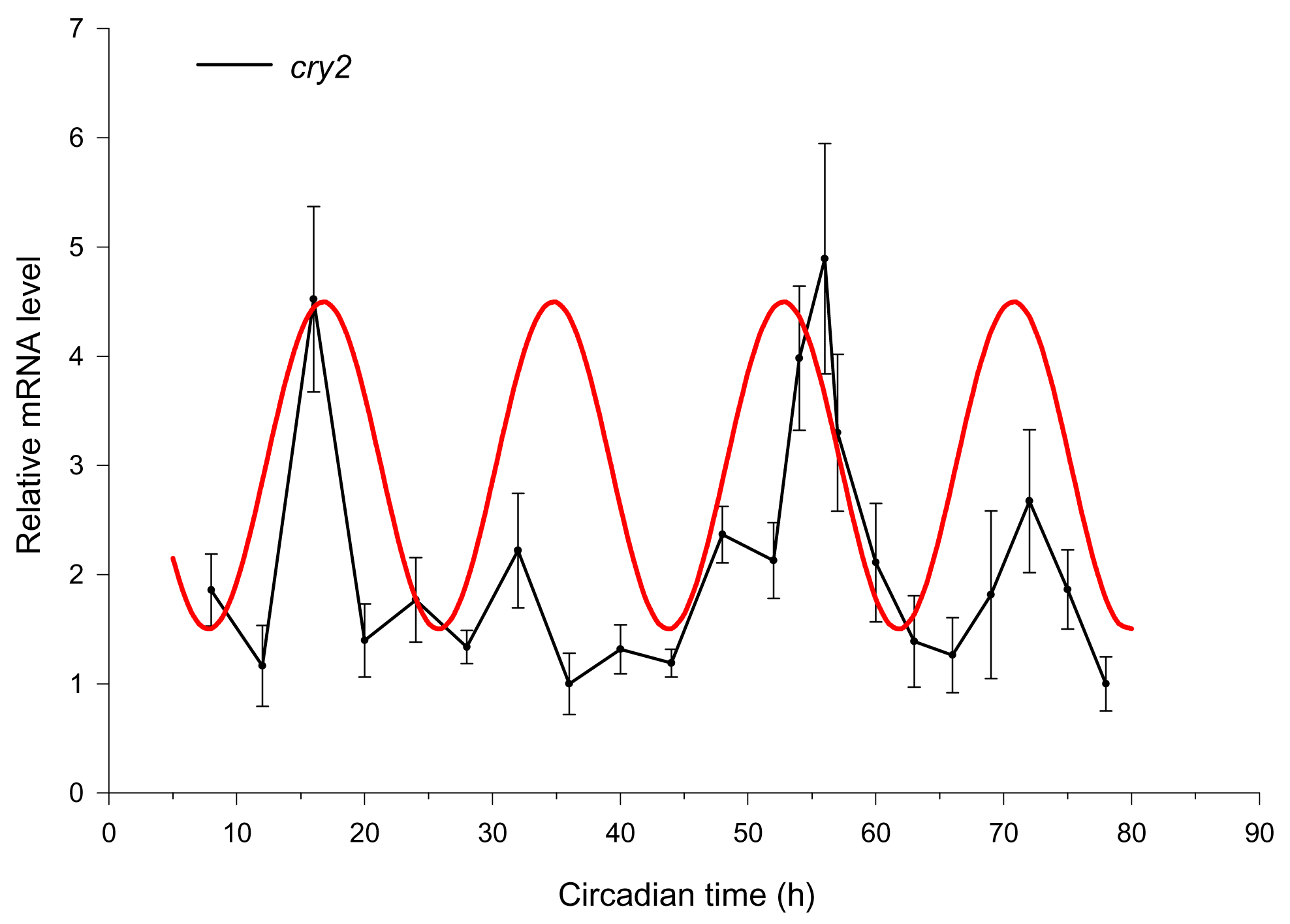

Supplement: Figure S1 — Antarctic krill exhibit a surprisingly short circadian period. Cry2 expression levels, monitored over 78 hours (combined data from two independent time courses in 2008 and 2010), reveal a significant circadian periodicity with a period of ∼18 hours (p<0.05, CircWave see Material and Methods). Predicted peaks from a fitted 18 h sinusoid (at circadian time (CT) 17, CT 35, CT 53, and CT 71) are correlating well with peak expression of cry2 mRNA level in vivo (at CT 16, CT 32, CT 56, and CT 72). (TIF) [file pone.0026090.s001.tif]
